# OLDER ADULTS AND CAREGIVERS’ ATTITUDES TOWARD MOBILITY AND IN-HOSPITAL MOBILITY RELATIONSHIPS

**DOI:** 10.1093/geroni/igae098.1792

**Published:** 2024-12-31

**Authors:** Anna Zisberg, Efrat Shadmi, Maayan Agmon, Yuliana Smichenko, Ksenya Shulyaev, Nurit Gur-Yaish

**Affiliations:** University of Haifa, Haifa, Hefa, Israel; University of Haifa, Haifa, Hefa, Israel; University of Haifa, University of Haifa, HaZafon, Israel; University of Haifa, Haifa, Hefa, Israel; University of Haifa, Haifa, Hefa, Israel; Center of Research & Study of Aging, University of Haifa; Oranim, Academic College of Education, Israel., Haifa, HaZafon, Israel

## Abstract

Improving mobility among hospitalized older adults remains a challenge. Qualitative research indicates that patients’ attitudes are one of the key factors associated with mobility levels. According to the Family Centered Approach, family members have an influence on both patients’ attitudes and actual mobility that are yet to be supported empirically. We tested whether inpatients and their family-members’ attitudes play a role in their in-hospital mobility. Paired data on in-hospital Attitudes Toward Mobility (ATM) was collected from patients (N=151; 78.6±6.6) and their family-caregivers (N=151; 58.2±14.2) as part of the larger prospective HoPE-MOR (Hospitalization Process Effects on Mobility Outcomes and Recovery) study. Data about patients’ health, functional, physical and mental status was collected at hospital admission. Data on average number of steps was collected during up to 7-days of hospitalization (Actigraphy). Results indicate that patients and caregivers’ ATM tend to be positive (3.6±.77; 3.7±.71) and patients were relatively mobile with average 1577±1328 daily steps. In a multivariate analysis of the factors related to average number of steps during hospitalization, both patients (β=.18, p=.02) and caregivers ATM (β=.15, p=.04) were significant predictors after accounting for patients’ mobility ability, severity of the acute and chronic illness, fear of falls, age and sex. In addition, caregivers’ ATM (β=.16, p=.04) was one of the main predictor of patients’ ATM, controlling for intervening variables. To conclude, there is an interlink between family caregivers’ attitudes, patients’ attitudes and actual patients’ mobility. Interventions should consider educating patients and their families to improve perceptions and attitudes toward mobility during hospitalization.

